# Hyperkinetic Gallbladder Syndrome: A Retrospective Study

**DOI:** 10.7759/cureus.91145

**Published:** 2025-08-27

**Authors:** Jose L Mejia, Luis A Mejia Sierra

**Affiliations:** 1 Surgery, WellSpan Ephrata Community Hospital, Ephrata, USA; 2 Vascular Surgery, Northwell Health, Long Island, USA

**Keywords:** acalculous gallbladder, chronic cholecystitis, elevated hida scan result, gallbladder dysmotility, hyperkinetic gallbladder

## Abstract

Gallbladder dysmotility issues present a significant challenge for clinicians and general surgeons. Adding a hepato-biliary scan to the diagnostic toolkit has greatly helped in understanding this condition, but how to proceed when dealing with hypermotility remains a real challenge, as predicting the outcome of a cholecystectomy, due to limited research, is difficult. Patients typically come to the office after an extended workup, with ultrasound and CT scans showing no abnormalities, normal liver chemistries, and an abnormal ejection fraction. At this point, the surgeon must clarify the plan and manage the patient's expectations.

A retrospective review was conducted of all cases encountered by a single surgeon over a two-year period in patients who presented to the office with biliary-type symptoms, normal ultrasound results, and an elevated ejection fraction on hepatobiliary iminodiacetic acid (HIDA) scan. The problem was classified as Hyperkinetic Gallbladder Syndrome (HGS) and given a grade. The primary goal was to identify pathological abnormalities in the collected specimens and to relieve symptoms.

Among all the laparoscopic cholecystectomies performed during this period, 32 cases with symptoms, negative ultrasounds, and elevated ejection fractions were identified. The male-to-female ratio was 1:4 (81% female patients), the median age range was 41-50 years, and 23 out of 32 patients had an ejection fraction above 80%. All removed specimens showed chronic cholecystitis in the histopathologic examination, and 30 out of 32 patients experienced symptom resolution at two to three weeks of follow-up.

HGS is a legitimate condition, and when patients are properly evaluated and other causes for their symptoms are carefully ruled out, laparoscopic cholecystectomy can alleviate the symptoms. It is crucial to select patients appropriately and set clear expectations.

## Introduction

Nearly 700,000 cholecystectomies are performed annually in the United States, with an estimated total cost of $6.5 billion [1]. The most common condition related to gallbladder disease is cholelithiasis [2-4]. Only 20% to 30% of patients with stones develop symptoms within 20 years; therefore, prophylactic cholecystectomy is not necessarily recommended for all of them [5]. However, there is another group of patients, those with biliary dyskinesia [6,7], who present with similar symptoms, normal ultrasounds, and abnormal hepato-biliary scans, which can make deciding the best treatment challenging. Generally, this condition is associated with a low ejection fraction (EF) on the hepatobiliary iminodiacetic acid (HIDA) scan, below 35% or 40%. Yet, it is not uncommon to encounter patients with elevated EFs [8-11], above 40%, and this group remains poorly explored and understudied. The gold standard treatment for symptomatic patients who can tolerate surgery is laparoscopic cholecystectomy. In the United States, this is the most common procedure performed by general surgeons. Most of these surgeries are due to cholelithiasis, while the remaining cases are classified into various categories, such as acalculous cholecystitis, dysmotility, and malignancy.

## Materials and methods

For this study, 32 patients with gallbladder disease symptoms were identified from 2018 to 2020 at Premier Surgical Associates, a private practice in Knoxville, Tennessee. The same surgeon performed the evaluation and procedures, and a retrospective chart review was conducted.

The inclusion criteria were negative ultrasound results, normal chemistries, and hepatobiliary scans with EFs above 40%. Any patients with cholelithiasis, or "sludge," present on ultrasound were excluded.

Appropriate history-taking and physical examinations were performed. The common complaints resembled those of gallstone disease, including intermittent right upper quadrant or epigastric pain, radiation to the right back, nausea, and occasional vomiting, often triggered by food.

Two primary endpoints were targeted: first, confirmation of abnormal histopathology, and second, correlation with symptom resolution. Patients were informed that, although limited research exists to predict outcomes, our opinion was that surgery might resolve symptoms in over 60% to 70% of cases. There was a 20% to 30% chance that symptoms would not fully resolve, and in 2% to 4% of cases, no resolution of the problem would occur, thereby establishing an expectation parameter.

IRB-equivalent approval was obtained from the medical director of this private group. The patients were verbally informed that their evaluation and treatment might be used for research purposes. A search of the practice database was conducted to match medical records to the diagnosis, initial visit, postoperative pathology report, and symptom resolution or persistence. To standardize hepato-biliary scan reports, we categorized them as Type I for patients with EFs ranging from 40% to 60%, Type II for those with EFs between 61% and 80%, and Type III for values above 80%. The pathology was designated as “Hyperkinetic Gallbladder Syndrome,” or HGS.

All patients underwent laparoscopic cholecystectomy [12], with follow-up scheduled two weeks postoperatively. Post-surgery, they received three days of Toradol, unless contraindicated for nonsteroidal anti-inflammatory drug (NSAID) use.

## Results

**Figure 1:**
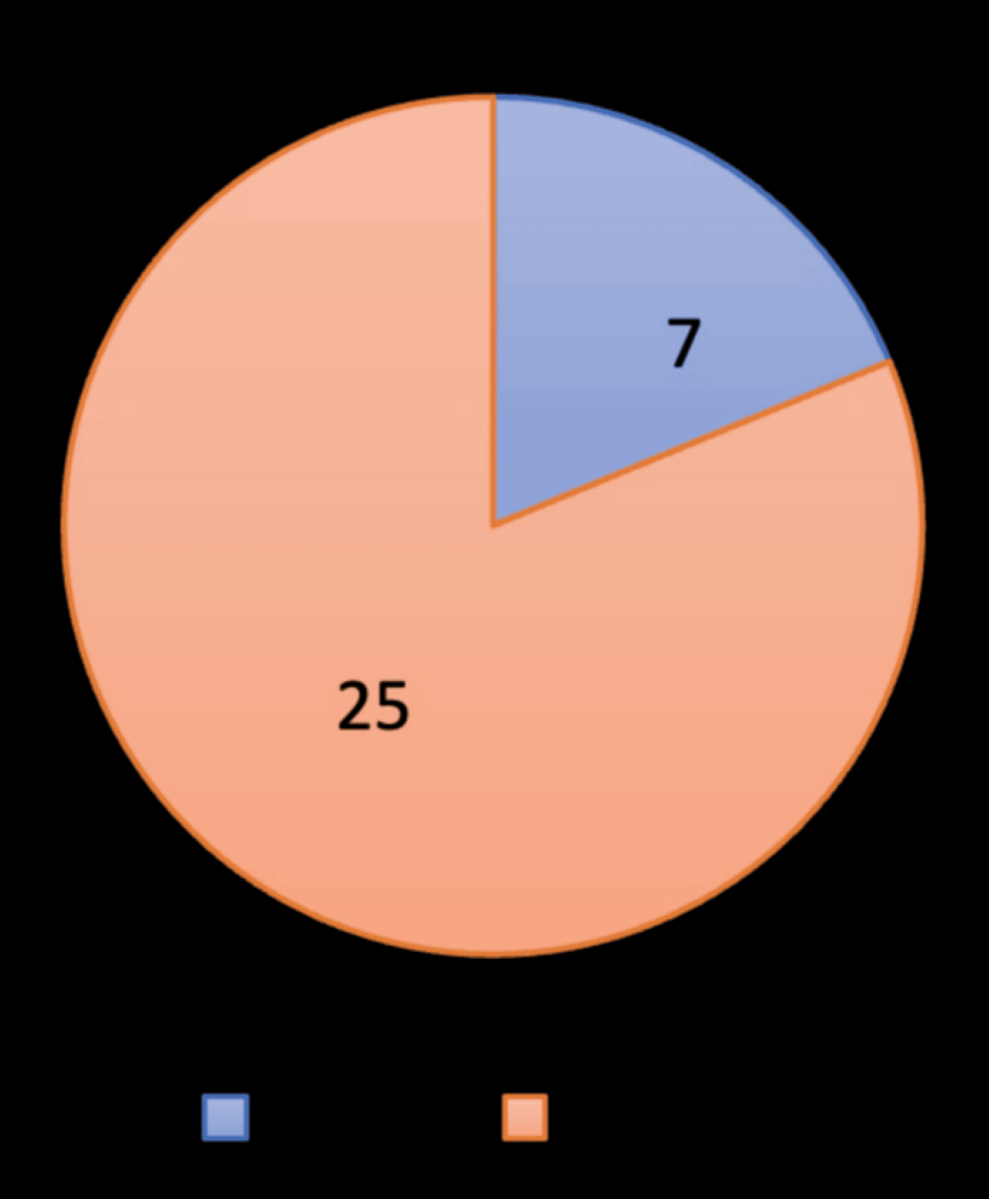
Gender chart Orange denotes 25 female patients; blue denotes 7 male patients.

A total of 32 patients were included in the study, comprising 25 females (78.1%) and 7 males (21.9%) (Figure 1), reflecting the established female predominance in gallbladder pathology.

**Figure 2:**
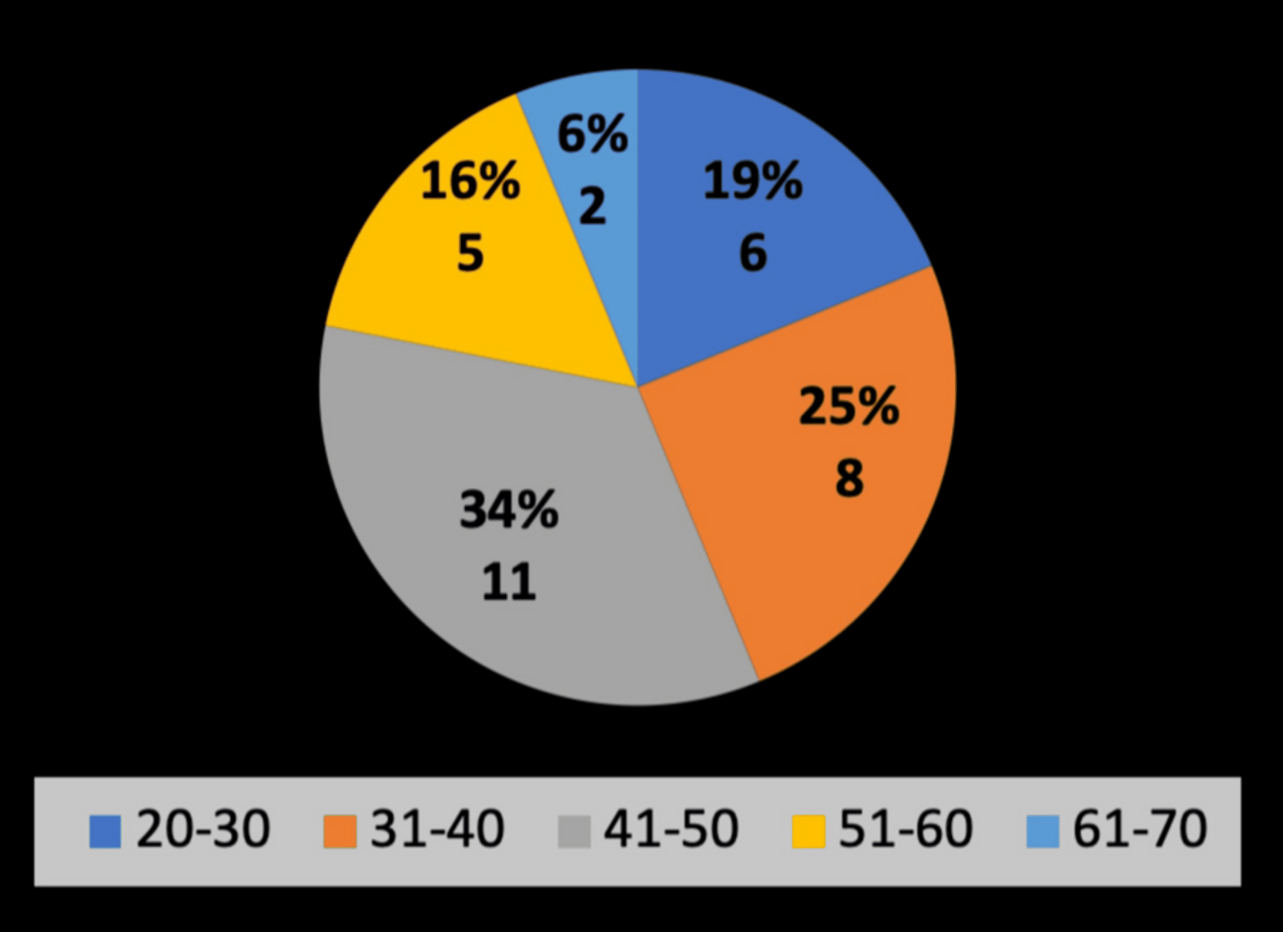
Age groups and percentages

The age distribution of patients was as follows (Figure 2): two patients (6.3%) were aged 61-70 years; five patients (15.6%) were aged 51-60 years; 11 patients (34.4%) were aged 41-50 years; eight patients (25.0%) were aged 31-40 years; and six patients (18.8%) were aged 20-30 years. These findings indicate that the most significant proportion of patients fell within the 41-50 year age range, with the majority (59.4%) between 31 and 50 years of age.

**Figure 3:**
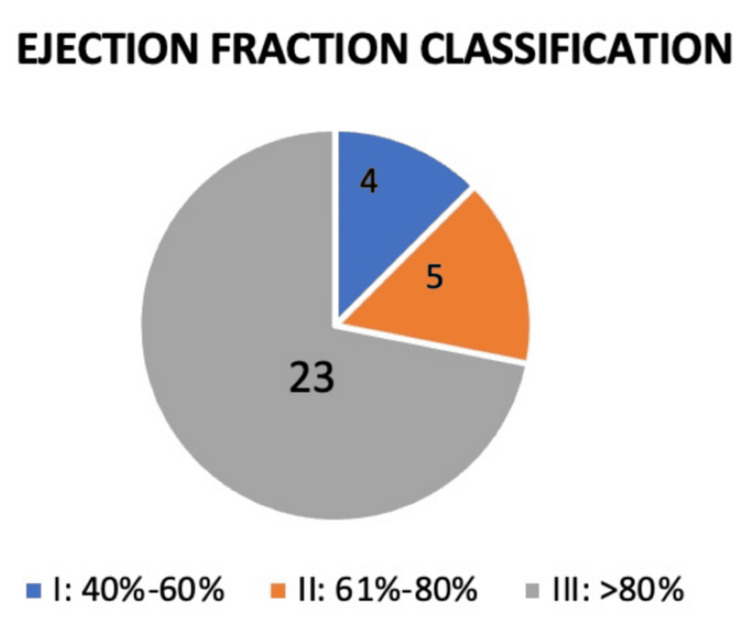
Ejection fraction classification Type I included 4 patients (blue), Type II included 5 patients (orange), and Type III included 23 patients (grey).

In terms of hepatobiliary scan findings (Figure 3), patients were categorized based on gallbladder EF as follows: Type I (EF 40-60%): four patients (12.5%); Type II (EF 61-80%): five patients (15.6%); and Type III (EF >80%): 23 patients (71.9%). The predominance of Type III scans, characterized by elevated gallbladder EF, is notable. This distribution supports emerging clinical observations that hyperkinetic gallbladder function may be associated with biliary-type symptoms.

At the two-week postoperative follow-up, histopathological examination of the gallbladder specimens revealed chronic cholecystitis in 100% of cases (n = 32). Clinically, 30 out of 32 patients (93.8%) reported complete resolution of preoperative symptoms, suggesting a high rate of therapeutic benefit from cholecystectomy in this patient population, including those with elevated EF.

## Discussion

HGS is increasingly recognized as a valid clinical entity within the spectrum of functional gallbladder disorders. When patients are subjected to a comprehensive diagnostic evaluation - one that systematically excludes alternative causes of abdominal pain - laparoscopic cholecystectomy has been shown to provide significant symptomatic relief and improvements in quality of life for appropriately selected individuals.

However, the success of surgical intervention in this pathology is highly contingent upon rigorous patient selection. An elevated gallbladder EF (EF >80%) alone should not be regarded as the sole indication for cholecystectomy. Instead, clinicians must adopt a multifactorial diagnostic approach, incorporating a detailed clinical history, reproducible symptom patterns consistent with biliary colic, physical examination findings, and corroborative imaging and functional studies. This integrated strategy helps to avoid unnecessary surgical procedures and ensures that operative management is reserved for those with the highest likelihood of benefit.

Moreover, preoperative counseling and shared decision-making are essential components of ethical, patient-centered care. Patients should be thoroughly informed of the potential benefits, expected recovery course, and the possibility - albeit low - of persistent symptoms despite surgery. Clear communication of these expectations fosters trust and aligns treatment goals with patient values.

Although the literature on gallbladder hyperkinesis remains limited, the available case series and retrospective reviews offer encouraging outcomes. For instance, Melvin et al. (2017) reported a strong correlation between elevated EF on hepatobiliary scans and symptom resolution following cholecystectomy in a subset of patients with hyperkinetic biliary dyskinesia [8]. Similarly, Bates et al. (2019) highlighted the diagnostic challenges posed by this syndrome [13], emphasizing that patients often present with normal imaging, yet demonstrate significantly elevated EF and favorable postoperative outcomes. These findings collectively support the notion that functional abnormalities, rather than anatomical defects, can be the primary drivers of biliary pain in select patients.

We recognize that this is a very limited study due to the small sample size and the short follow-up period of two weeks. However, as we limited the goals of the review to the resolution of symptoms and correlation with pathology, we believe the results found are indicative of an improvement in quality of life. We hope that, at some point in the future, we can expand the review with a larger sample and more extensive follow-up at 6 and 12 months.

In summary, HGS should be approached as a functional gastrointestinal disorder requiring individualized assessment and management. The absence of standardized diagnostic criteria underscores the need for further prospective studies to better characterize the pathophysiology, refine diagnostic algorithms, and establish evidence-based treatment protocols. Until then, the combination of clinical vigilance, diagnostic thoroughness, and transparent communication remains the cornerstone of effective care for patients with this emerging condition.

## Conclusions

This review highlights that in appropriately selected patients, cholecystectomy can yield positive outcomes by addressing a functional abnormality - namely, a hypercontractile gallbladder - that may cause postprandial right upper quadrant pain, nausea, and other dyspeptic symptoms, even in the absence of structural pathology. The targeted intervention emphasizes understanding the underlying motility disorder and recognizing its role in symptom generation, thereby providing a more precise treatment approach for patients with suspected biliary dyskinesia.

Ultimately, gallbladder dysmotility, particularly in patients with elevated EF on HIDA-CCK (cholecystokinin) imaging, can mimic classic biliary colic despite lacking gallstones or wall abnormalities. The exaggerated contractile response may lead to transient pressure increases, dyskinesia, or localized inflammation, contributing to chronic discomfort. When accurately diagnosed and other causes are ruled out, cholecystectomy can be a beneficial and potentially reversible treatment option, reflecting the growing recognition of functional gastrointestinal disorders and the importance of personalized, physiology-based surgical care.
